# Main factor causing “faster-is-slower” phenomenon during evacuation: rodent experiment and simulation

**DOI:** 10.1038/s41598-017-14007-6

**Published:** 2017-10-20

**Authors:** Hyejin Oh, Junyoung Park

**Affiliations:** 1 Department of Mechanical Design Engineering, Graduate School, Kumoh National Institute of Technology, 61 Daehak-ro, Gumi, Korea; 2 Department of Mechanical Design Engineering, Kumoh National Institute of Technology, 61 Daehak-ro, Gumi, Korea

## Abstract

Understanding crowd flow at bottlenecks is important for preventing accidents in emergencies. In this research, a crowd evacuation passing through a narrow exit connected with guide-walls is analysed using the discrete element method based on physical and psychological modelling in parallel with empirical rodent research. Results of rodent experiment and simulation demonstrate the faster-is-slower (FIS) effect, which is a well-known phenomenon in pedestrian dynamics. As the angle of the guide-walls increases, agents rapidly evacuate the room even though they have low velocity. The increase in this angle causes agents to form lanes. It is validated that ordered agents evacuate expeditiously with relatively low velocity despite expectations to the contrary. The extracted experimental and simulation data strongly suggest that the agents’ standard deviation of velocity can be a key factor causing the FIS effect. It is found that the FIS effect can be eliminated by controlling the standard deviation.

## Introduction

Panic, defined as an extremely stressed condition caused by an emergency state, makes people push and shove each other for their own safety. The force generated within a crowd is estimated to be at least 4,450 N; this formidable force is enough to trigger fatal accidents^1^. The Kiss nightclub fire of Santa Maria in Brazil is a notable example^2^. When the fire broke out, a crowd of over 2,000 rushed toward the nightclub’s only exit, and 231 people were crushed and killed. Note that such an accident may not be caused by actual danger such as fire or earthquake. For instance, in Sangju Stadium, South Korea, 11 people died because of an excited crowd attempted to enter the concert hall through a single entrance that was suddenly opened during a rehearsal^3^. Likewise, in 2010, there were more than 500 casualties at the love parade disaster in Germany as the vast crowd became jammed at a single tunnel^4^. These cases are similar regardless of whether there was real danger: a high level of anarchy filled the limited and densely packed space, even if there were police officers and members of staff endeavouring to direct people. Thus, an appropriate strategy based on an accurate understanding of this phenomenon is needed. Research, such as on the correlation of crowd scale with building structure and what actually happens between them, is needed. Therefore, numerous studies have been conducted on crowd flow, mostly by simulation.

Research based on the cellular automata (CA) model and social force model are well known in pedestrian dynamics. The CA model, especially, is generally used for its computational simplicity. According to Daoliang^5^, the critical values are the width, number of exits, and distances between two exits. Moreover, research on the obstacles near exits^6^ and kin behaviour^7^ has found that various building structures and pedestrians’ psychological features could be significant indicators of crowd flow. Despite its numerical advantages, the cell-based CA model does not consider collision among pedestrians does not express clogging at an exit because individuals occupy empty cells and constantly pass through the exit. In addition, there is the criticism that the CA model cannot yet express detailed personal autonomy perfectly^8,9^. Meanwhile, Helbing^10^ suggested a social force model that treats pedestrian flow as a many-particle system. In this model, forces acting on each individual are divided into physical and socio-psychological forces and a faster-is-slower (FIS) effect appears in crowd evacuation. However, the social force model is estimated to not be fully consistent with empirical observations, and might be difficult to calibrate at time^11^. Detailed considerations regarding the limitations of such numerical models are summarized by Was^12^.

There are several modified versions of the social force model. The discrete element method (DEM) with psychological rules is one of them. DEM analyses the movement of granular materials such as chemical or metal powders and grain, and DEM is mostly used in pharmaceutical, agriculture, and material processing industries^13–15^. Based on this method, each pedestrian is treated as a particle following psychological rules, and particle collisions are calculated as a spring-dashpot system. This method is a more realistic way to express actual jamming, clogging, and crashes within the crowd because, unlike the social force model, which calculates the contact force among pedestrians virtually, DEM considers the contact force based on real physical force. It is essential to express realistic contact force because according to current trends, collisions are inevitable in an emergency evacuation. Calculating the physical force acting on each pedestrian particle enables us to predict the number of casualties that could occur in various circumstances. Some modified DEM models also exist, specifically, the Tsuji, Gotoh, and Langston models. Although these models were derived from DEM, each model follows similar but slightly different psychological rules. In particular, Ueda^16^ enhanced the Tsuji model to simulate various types of flows: one-way (emergency evacuation), two-way, and cross-way. However, the Gotoh and Langston models are optimized for bidirectional pedestrian flow in complex building structures^17,18^. In addition, there is no emergency evacuation model in the Gotoh and Langston models. The Tsuji model not only effectively expresses the psychological effects in densely crowded people by applying several psychological rules to DEM, but also has been applied to real casualties. In 2003, the crushing deaths accident at Akashi Bridge in Kobe, Japan, was successfully simulated using the Tsuji model^19^. In addition, Song and Park^20^ used the same model and reported that when people evacuate through an abruptly curved corridor, more accidents could occur at the corner. The Tsuji model fits relatively well with theory compared with the other two models in the case of highly dense circumstances^21^. Given that this research focuses on a crowd emergency evacuation and analyses phenomena among the crowd near an exit, the Tsuji model yields the most appropriate results.

As previously mentioned, numerous researchers have studied pedestrian flow in various circumstances and various phenomena such as arching, self-queuing, jamming, and clogging have been observed in the research. To compare our simulation and experimental results, we focus on research that demonstrates the FIS effect. Experimental results exist wherein humans and sheep have shown the FIS effect^22^. However, research including both simulation and experiment data under the same conditions is very rare because it is difficult to express experimental parameters numerically. In addition, several results in these studies have been represented using power-law relations for complex systems. In complex systems, non-linear phenomena interact with each other, showing linearity macroscopically, which is somewhat ambiguous. Therefore, a more intuitive parameter is necessary to understand the essence of the FIS effect precisely and to compare simulation and empirical data.

Normally, the FIS effect is induced by the impatience of distressed people. According to Helbing, this impatience is closely related to an increase in their desired velocity^10^. When people try to move faster, the average speed of exiting decreases. Moreover, distressed people introduce a panic factor, which represents the degree of herding behaviour of a pedestrian. The instant herding behaviour of pedestrians causes clogging at an exit or a bottleneck and could lead to fatal accidents. However, it is interesting that the proper degree of panic can be a successful solution for effective evacuation. The FIS effect and clogging at narrow exits are not well understood yet owing to their complexity. Therefore, numerous researchers have conducted simulations to reveal why the FIS effect occurs and what happens at the bottleneck during an emergency, using various numerical methods and experiments. Helbing described oscillations at bottlenecks^23,24^, and Lakoba *et al*. and Parisi focused on the FIS effect^25,26^ using the social force model. In addition, Perez *et al*.^27^ observed the FIS effect using a CA model that utilizes the level of anxiety or panic. Moreover, several experimental researches investigated pedestrian flow at bottlenecks^28,29^. Unfortunately, research dealing with these problems employing both simulation and experimental methods, especially in highly urgent situations, is scarce because an experimental approach involving humans is difficult to control.

However, in several studies, people have participated in evacuation experiments. Chen *et al*. conducted an experiment concerning the evacuation from a four-story building in different conditions, targeting young university students^30^. Proulx designed an evacuation experiment for apartments with 6–7 stories^31^. Further, a study presented data concerning the evacuation of older adults and people with mobility impairments, providing adequate value for other researches^32^. However, these experimental studies did not address the FIS effect. Notwithstanding, Garcimartin *et al*. conducted evacuation experiments at a bottleneck to observe the FIS effect^33^; the authors mentioned that only small variations of desired velocity were allowed because the experiment involved the high risk of real-life casualties, as mentioned before. This limitation means that most experiments deviate from real situations, which are extremely distressing and dangerous.

As an alternative, recent experiments have involved non-humans such as fish, woodlice, sheep, and mice. Saloma *et al*. conducted mice experiments using water for fear-conditioning^34^; however, this is a different method compared to the one employed in this research as they did not consider evacuation on land. Moreover, the approach to determine the causes of the FIS effect was not mentioned. Research using hungry sheep, which appears to be more similar to humans than mice^35^, did not analyse the fundamental reasons for the occurrence of the FIS effect. A more realistic experiment could be conducted by considering that fear created from aversive stimuli can be a more powerful motivation for evacuation than food. Other studies involving fish and woodlice are not comparable with ones involving humans because of the difference in species^36,37^. Therefore, in this research, experiments using mice as well as simulations regarding the evacuation flow in panic-inducing situations are compared and analysed to reveal the main cause of the FIS effect. Here, the advanced Tsuji model is adopted as an evacuation model. Further, some phenomena that could occur during the evacuation are observed and a possible cause is examined.

Mice are especially of interest as they have two cerebral parts, an amygdala and hippocampus, which control emotions of depression and fear, respectively, as do humans^38^. Additionally, several conventional fear-conditioning tests, including an active avoidance test and a water maze test, help experiments about skill to be conducted^39^. Strictly speaking, to apply simulation results based on animal experiments to humans quantitatively might be difficult at the moment because there is no definite evidence for the analogy between human and mouse behaviour. It is obvious that humans and mice have different movement mechanisms. In addition, for people, the source of danger during a bottleneck, such as a fire in an emergency situation, is normally located behind them, while the source in the mouse experiment in this study is electric current located on the floor. However, one can qualitatively presume human behaviour through this work based on some supporting studies. Nevertheless, although humans are more susceptible to the environment because they exist in highly structured spaces consisting of all sorts of obstacles, animal groups still exhibit a complex organization of the whole group^40^. Based on this self-organization ability, crowd and animal motions have been modelled as intelligent particles that have decision mechanisms: attraction, repulsion, and alignment^41^. However, under the certain conditions, particles without intelligence exhibit a “self-organization and pattern formation” that is the same as intelligent particles, even though they do not have the decision mechanisms^42,43^. In this context, the relationship between mice and humans is closer than it is between intelligent particles and non-intelligent particles because mice show self-organization behaviour^36^ based on their intelligence. The difference between humans and mice is just the extent of intelligence, not whether intelligence exists or not. Unfortunately, there is no solid study that identifies the difference or analogy between human and animal behaviour pertaining to panic. However, we can infer the similarity between human and animal behaviour even in panic because they both share identical phenomena such as FIS and self-organization^44,45^. In this research, we suggest a fundamental approach to human crowd evacuation with reference to mice experiments and a simulation that is based on the experimental data.

## Results

### Comparison with existing research

**Figure 1:**
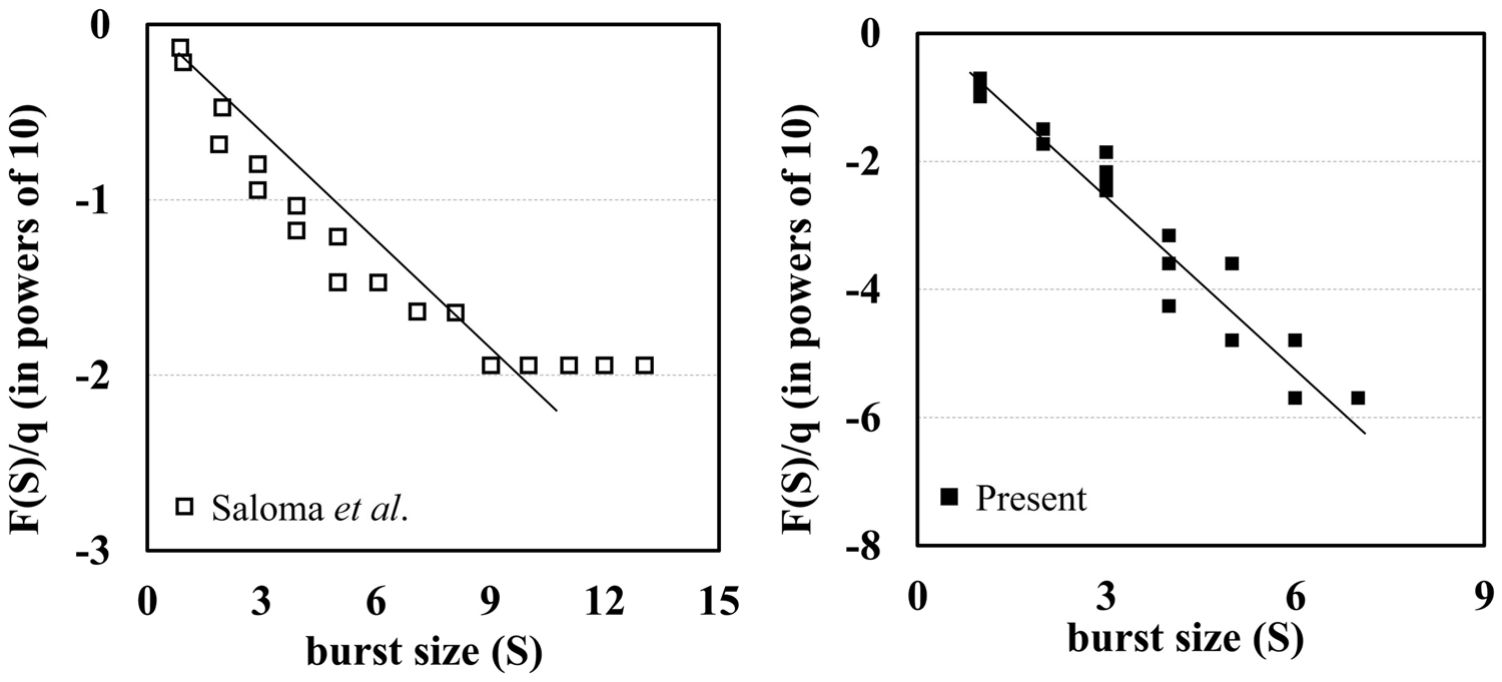
Linearity in the simulation with CA model and DEM.

Figure 1 shows the simulation results from the CA model^34^ and DEM under the same conditions. These two systems have a single-exit evacuation scenario and there are no guide-walls. When the frequency distribution $F(S)$ is represented by the power-law, burst size *S* and its frequency distribution $F(S)/q$ in powers of 10, where *q* is an iteration step, are linear in both results. This means that in a macroscopic aspect, the evacuation scale and its frequency have an inverse proportional relation as they do in the DEM simulation results. Based on this validation in the view of complex system, a closer observation near the various levels of bottleneck is discussed in the next section.

### Comparative analysis of mice experiment and DEM simulation

#### Qualitative observation

**Figure 2:**
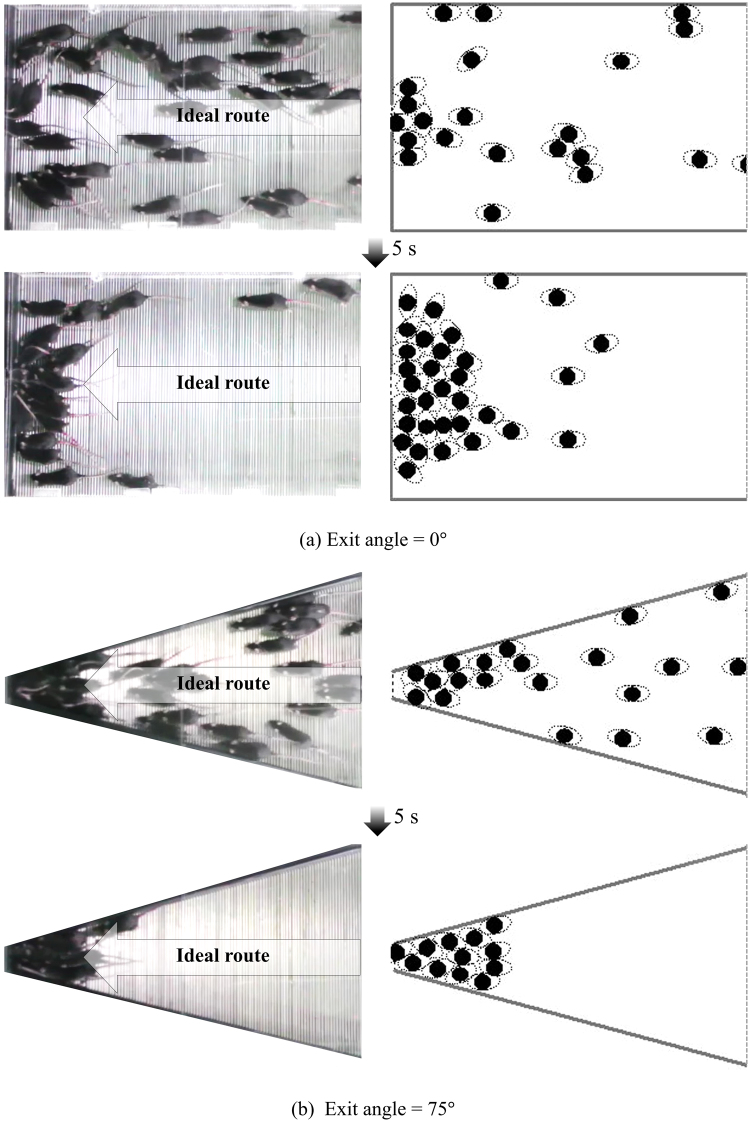
Snapshots of the experiment (left) and simulation (right).

Figure 2 shows snapshots of the experiment and simulation results. Basically, the mice proceed to the front from the location where they first enter. However, the mice do not always follow the ideal route. Some mice follow the ideal route toward the exit at the beginning, and some of the others seems to go forward without any decision-making such as repulsion, attraction, or alignment, and then take the ideal route towards the exit at a later moment. In addition, some mice also seem to go forward straight without any consideration for the environment such as an exit or wall, and then they head toward the exit right after they have encountered the exit wall. According to an empirical observation of video, about half of the mice can find their own ideal route. In addition, some mice often cause small-scaled herds by climbing on other mice to avoid the electric current in the experiment. However, we could not find any clear evidence for the herding behaviour of mice. In Fig. 2(a), some mice standing near the walls follow along the wall and reach both sides of corners next to the exit. After 5 s, these mice approaching from the sides interrupt the evacuation of the other mice following the ideal route. In the experiment, sometimes a situation occurs wherein two or three mice do not wait their turn and search for another way out. In contrast, in Fig. 2(b), all mice follow the path guided by walls of 75°, heading directly to the exit. Here, the mice are aware of each other and follow a lane-formation, which is a significant factor for an orderly and safe evacuation. Although a certain degree of jamming exists at the exit, they seem to notice that there is the only one exit and wait for their turn. In both cases, the experiment results are qualitatively quite well described by the simulation as seen in Fig. 2.

#### Quantitative analysis

**Figure 3:**
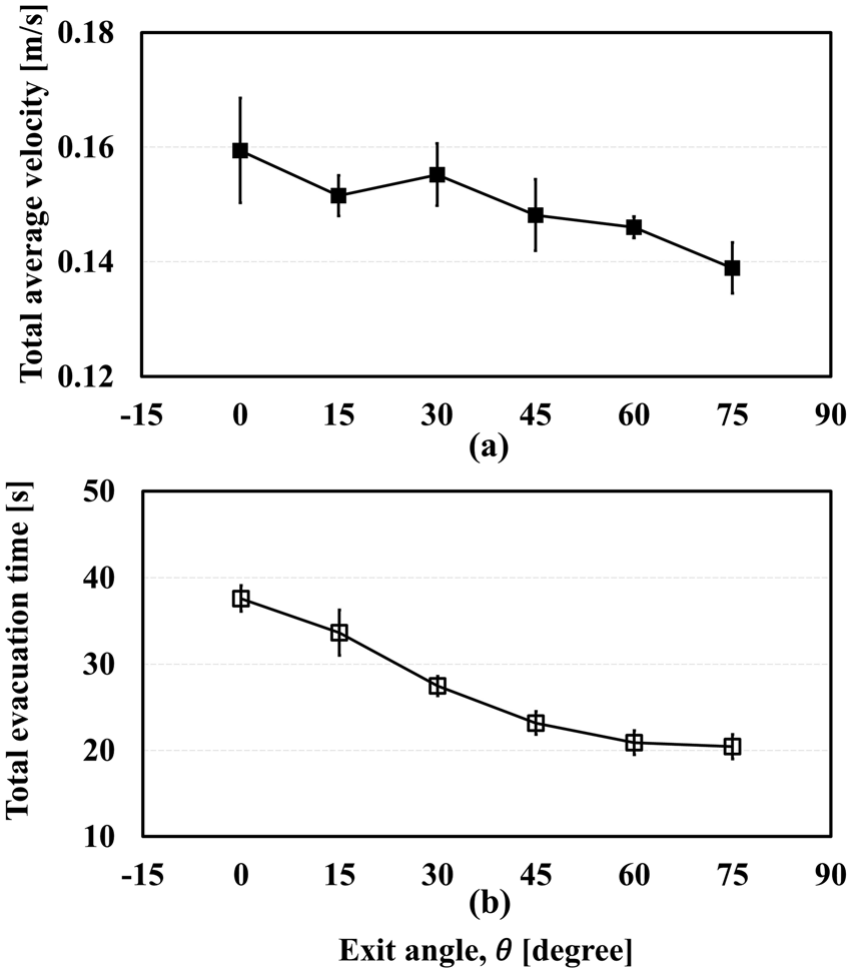
Experimental results; total average velocity (**a**) and total evacuation time (**b**).

As mentioned in Section 4.4, ${\bar{V}}_{total}$ and ${\bar{T}}_{evc}$ can be evaluated from the experiment videos, as depicted in Fig. 3. ${\bar{V}}_{total}$ declines as *θ* increases, whereas ${\bar{T}}_{evc}$ increases with increasing $\theta$ in general. When a few subjects evacuate in a spacious room, the higher velocity they have, the faster they leave the room because they do not disturb each other’s evacuation. However, as the number of subjects per area becomes larger, just like in this experiment, the high velocity of each subject could cause a delay in evacuation. This is because the space that the mice can utilize is sufficient when $\theta$ is zero; thus, they can select their own route. Although it makes for a high ${\bar{V}}_{total}$, the different routes could give rise to chaos at the narrow exit, increasing ${\bar{T}}_{evc}$. On the contrary, when $\theta$ is 75°, the evacuation route given to the mice is limited by the walls. This effective restriction leads ${\bar{T}}_{evc}$ to decrease even if ${\bar{V}}_{total}$ is low. These results indicate the FIS effect, suggesting that the guide walls influence the evacuation flow. To examine these results closely, we assume that the velocity distribution of mice in the lane formation pattern is smaller tat in a random formation.

**Figure 4:**
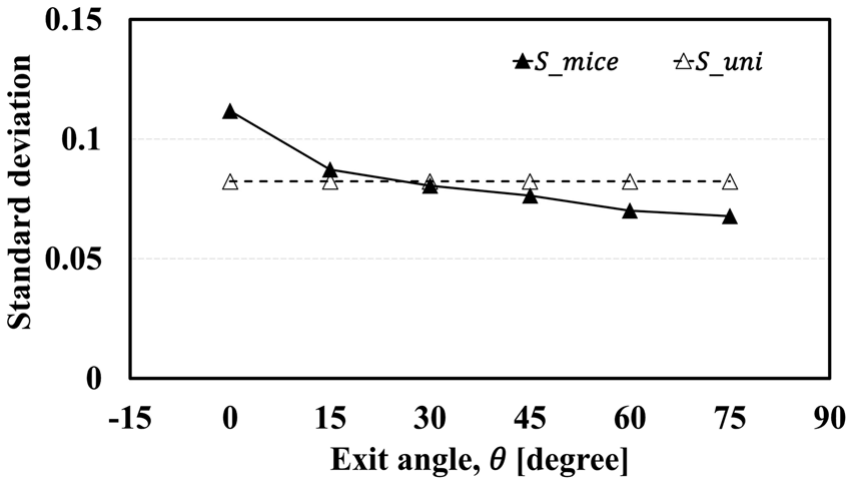
*S*_*mice*_ and *S*_*uni*_ along the exit angle.

According to the result shown in Fig. 4, ${S}_{mice}$ tends to decrease with increasing $\theta$, and it reaches a minimum when $\theta$ is 75°. That is, the velocity difference of each mouse heading for the exit is relatively big at low $\theta$, whereas it is small at high $\theta$. As we mentioned in Section 4.4, the value of ${S}_{uni}$ is uniform at all exit angles. Therefore, we apply the standard deviations of the mice to the DEM simulation in two ways to validate whether the standard deviation of pedestrians’ velocities influences the FIS effect.

**Figure 5:**
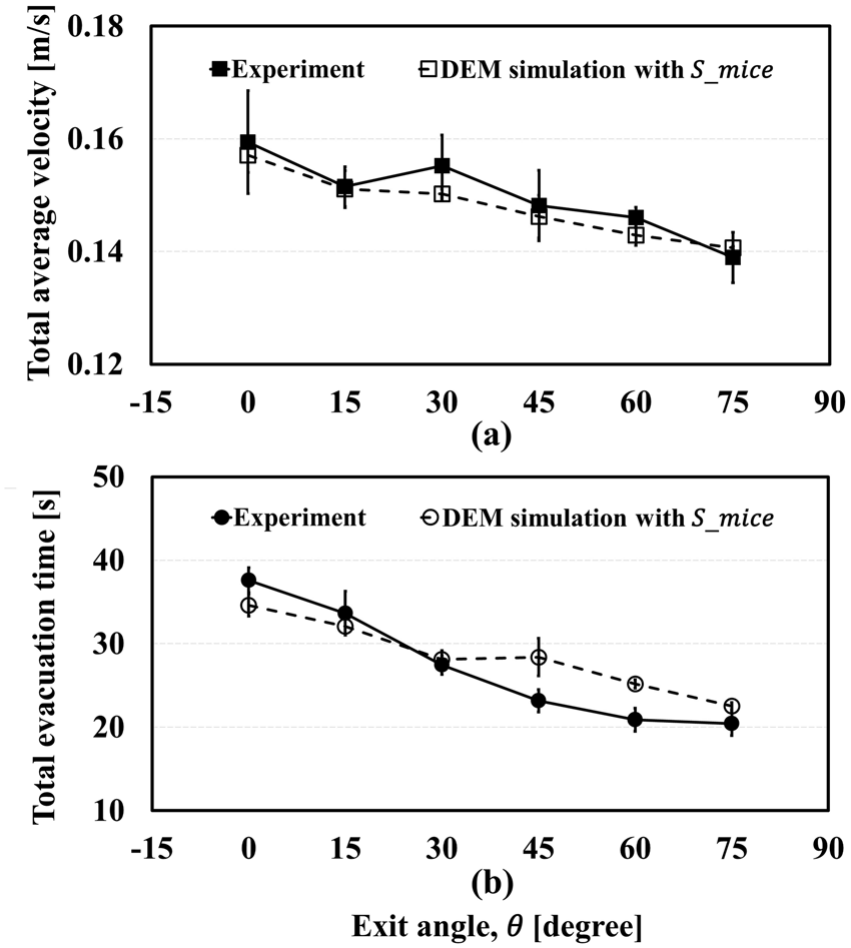
Results with varied standard deviation.

**Figure 6:**
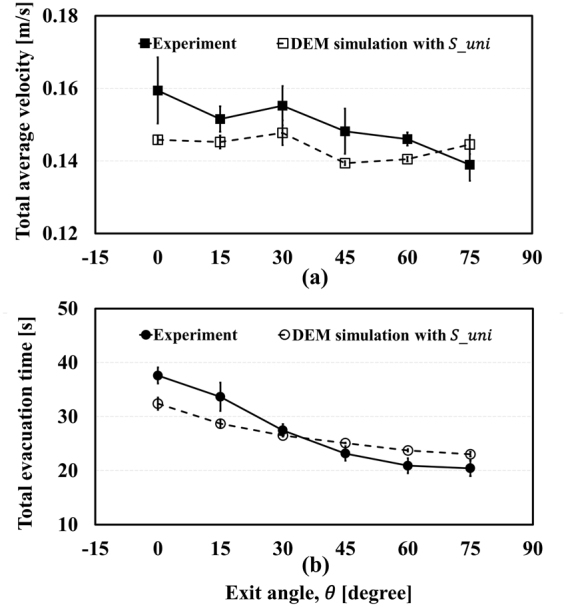
Results with uniform standard deviation.

**Figure 7:**
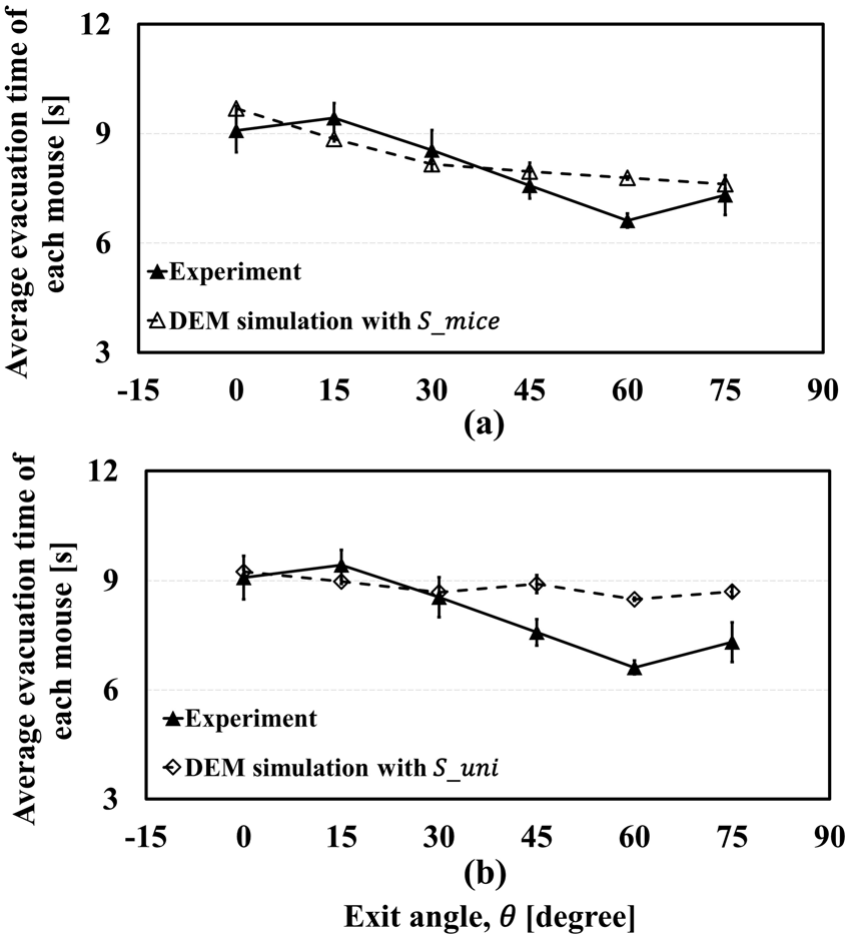
Effect of standard deviation on average time to complete evacuation of each mouse.

*S*_*mice*_ and ${S}_{uni}$ were applied to the evacuation simulation, as represented in Fig. 5 and Fig. 6, respectively. In Fig. 5, the simulation results show quite good agreement with experimental results. In addition, they also represent the FIS effect with ${S}_{mice}$ in Fig. 5. However, although ${\bar{T}}_{evc}$ still seems to fit with the experiment results, ${\bar{V}}_{total}$ appears to be independent of the change of exit angle, as shown in Fig. 6 with ${S}_{uni}$. In other words, manipulating the standard deviation of pedestrians’ velocity can remove the FIS effect. Clearly, it could be argued that these plots do not have a significant meaning because the total evacuation time is highest at 0° and lowest at 75° whether ${S}_{uni}$ and ${S}_{mice}$ are used or not. This is because, in general, the total evacuation time is a crucial factor for judging the success of an evacuation. To estimate casualties more accurately, however, total evacuation time is not the only factor to be considered, as shown in Fig. 7. This figure shows ${\bar{t}}_{evc}$, the averaged time taken to complete evacuation by each mouse when confined in the panicky space according to the exit angle, and it tends to be inversely proportional to the exit angle with ${S}_{uni}$. When a fire occurs, for example, there would be more casualties at low exit angles as pedestrians in this situation are exposed to the smoke for a long time. Even if one individual starts evacuation immediately, he/she could still be in danger owing to the long time taken to completevacuation because of the panic-fuelled and unordered situation. When ${S}_{mice}$ is applied, the simulation result follows the experimental result. In the simulation result with ${S}_{uni}$, the average evacuation time of each mouse does not decrease as the exit angle increases. Total evacuation time ${\bar{T}}_{evc}$ does not show obvious changes according to t standard deviation, but ${\bar{t}}_{evc}$, the average evacuation time of each mouse, suggests that the evacuation flow is clearly affected by the standard deviation of the velocity. From this point of view, additional experiments and simulations in which pedestrians’ velocities can be controlled should be conducted for detailed research regarding the FIS effect.

In reality, the FIS effect would be caused by the interactions of more complex factors. For now, approaches using other variables such as the number of pedestrians and diversity among species were not considered. However, we observed the FIS effect in experiments, anticipating that it is relevant to the pedestrian lane formation. Therefore, the standard deviation of pedestrian velocity related with lane formation was manipulated in the simulation. This could eliminate the effect, indicating that ${S}_{mice}$ could be a key factor of the FIS effect.

## Discussion

Our aim in this work is to presume pedestrian flow in the case of an emergency. The behaviour of people in such a real-life situation is not entirely known because conducting human experiments under threatening environments could lead to real casuales. However, we can compare the numerical simulation and mice experiment indirectly by assuming that the behaviour of mice can be regarded as human behaviour. For many years, several psychological rules have been applied to DEM for analysing pedestrian dynamics because of its effective algorithm for calculating contact among pedestrians. In this study, the evacuation experiments for 50 mice using an active avoidance test were conducted to validate the simulation model, and the FIS effect was observed by varying the angle of the guide walls. This effect was caused by lane formation in accordance with the increase in guide wall angle. In addition, the standard deviation of pedestrian velocity was confirmed to be one of the factors influencing this effect by comparing it with the DEM simulation. Specifically, if people evacuate from the emergency situation while maintaining order, they can evacuate rapidly with less effort. The DEM simulation could describe the evacuation flow, and this microscopic approach strengthens the basis for future work regarding other major factors tdetermine and control the effect.

## Methods

### Equations of motion for agents

DEM is an individual-based system. Although this is a similarity with the social force model, DEM places emphasis on actual crashes among the agents. The movement of each agent can be determined by the equations of motion for the agents starting with Newton’s second law.

1 $${d}^{2}\vec{r}/d\vec{t}=\vec{F}/m=({\vec{F}}_{contact}+{\vec{F}}_{psy})/m$$

In the equation above, $\vec{r}$ is the position vector of an agent, *m* is the mass of the agent, and $\vec{F}$ is the summation vector of forces acting on the agent. It represents the motion of the agent by dividing the force into contact force and psychological force because each agent can walk at will. Normally, an agent is represented by a circle or an ellipse in two-dimensional ow.

### Contact Force

**Figure 8:**
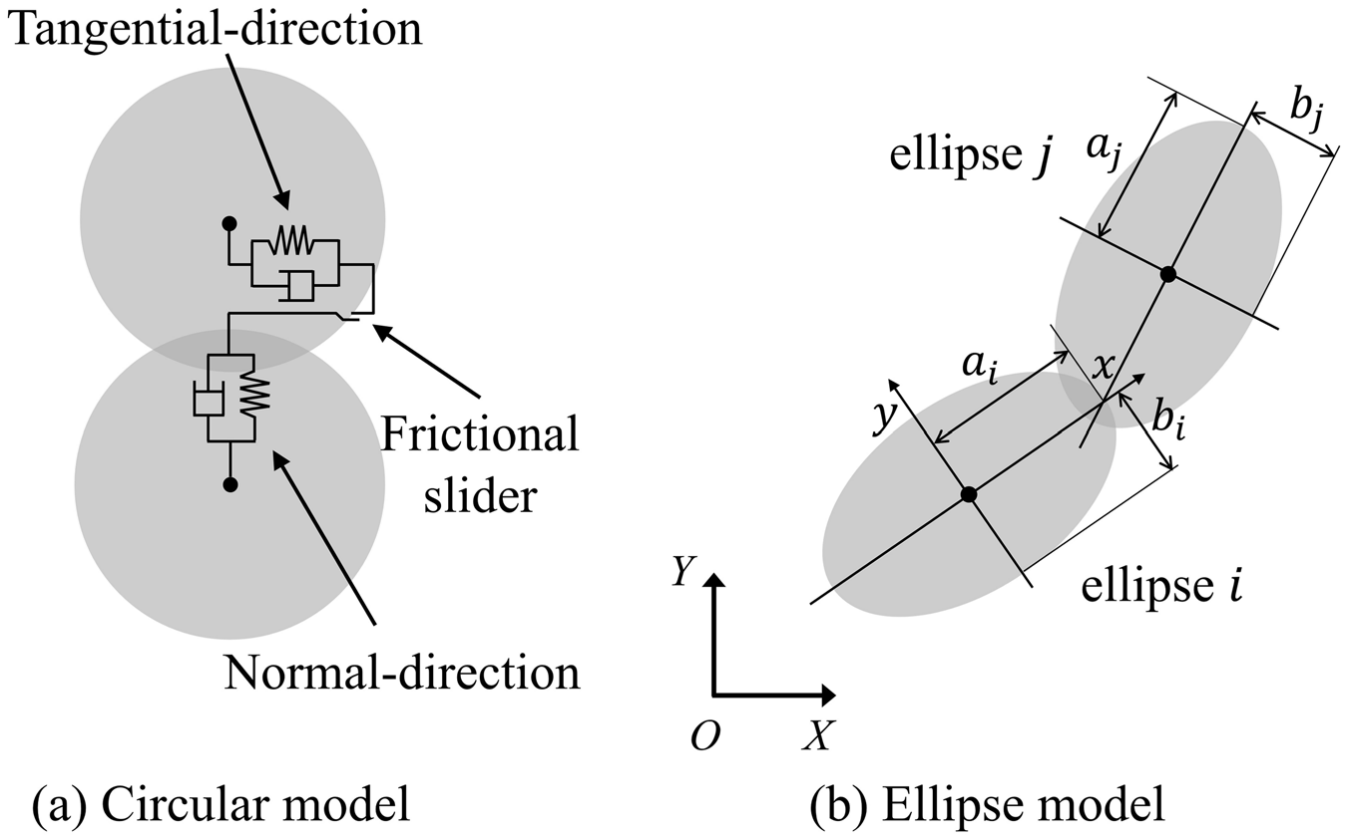
Schematic diagram of the linear spring and dashpot system.

Jamming among agents can be represented effectively by permitting a very small amount of overlap by adapting a soft-particle method^46^. The collision of the two pedestrians can be treated as the linear spring and dashpot system in Fig. 8. The contact force ${\vec{F}}_{contact}$ is given by

2 $${\vec{F}}_{contact}=(-k\delta -\eta {\vec{v}}_{r}\cdot \vec{n})\vec{n}$$

where *k* is the spring constant, ${\rm{\delta }}$ is overlap distance, ${\rm{\eta }}$ is damping coefficient, ${\vec{v}}_{r}$ is the relative velocity vector, and $\vec{n}$ is unit vector between agents. There are several ellipse models for simulating pedestrian flow. Was and Lubas^12,47^ simulated an ellipse-shaped pedestrian flow for various scenarios with the CA model. Chiraibi *et al*. simulated pedestrian flow at a bottleneck with the force-based ellipse model^48^. However, the fundamental concept of the CA model is different from DEM, and these models have their own limitations, as mentioned in the introduction. In addition, many ellipse models based on the DEM have been developed to analyse granular materials, and they have been enhanced in past years^49–52^. For these reasons, we implemented Ueda’s contact algorithm for a DEM ellipse model^23^ to calculate the overlap distance of two contacted elliptical particles, as shown in Fig. 8(b), starting with an ellipse equation in the local coordinates, as follows

3a $${(x/a)}^{2}+{(y/b)}^{2}-1=0$$

where *a* and *b* are the major and minor axes. The contact of two particles is determined by following equation in global coordinates:

3b $${c}_{4}{\bar{x}}^{4}+{c}_{3}{\bar{x}}^{3}+{c}_{2}{\bar{x}}^{2}+{c}_{1}\bar{x}+{c}_{0}=0$$

where $\bar{x}$ is $x/{a}_{i}$, other coefficients ${c}_{4}$, ${c}_{3}$, ${c}_{2}$, ${c}_{1}$, and ${c}_{0}$ substitute for complicated mathematical procedures and are a function of location and pure geometrical parameters, such as ${a}_{i}$, ${b}_{i}$, ${a}_{j}$, ${b}_{j}$ and so on. The details are fully described in Ueda’s paper. This contact algorithm, which calculates ${\rm{\delta }}$, is for a mouse model, and the other processes for obtaining ${\vec{F}}_{contact}$ are the same as in the circular model.

### Psychological Force

#### Pedestrian psychology in panicked state

The behaviour of an agent, such as a pedestrian or mouse, in an emergency situation can be affected by several psychological factors. In an extremely distressed state induced by external stimuli, agents tend to break for the narrow exit and generate a bottleneck flow. As agents attempt to escape more quickly than others, they do not generally maintain order and tend to not avoid possible collisions with others. The following are the behavioural conditions used in an emergency simution state to mimic real emergencies:

1. Rushing to the exit.
2. Overtaking other pedestrians walking in the same direction.
3. Stepping away from the nearest wall.
4. Avoiding other pedestrians walking at their side.

**Figure 9:**
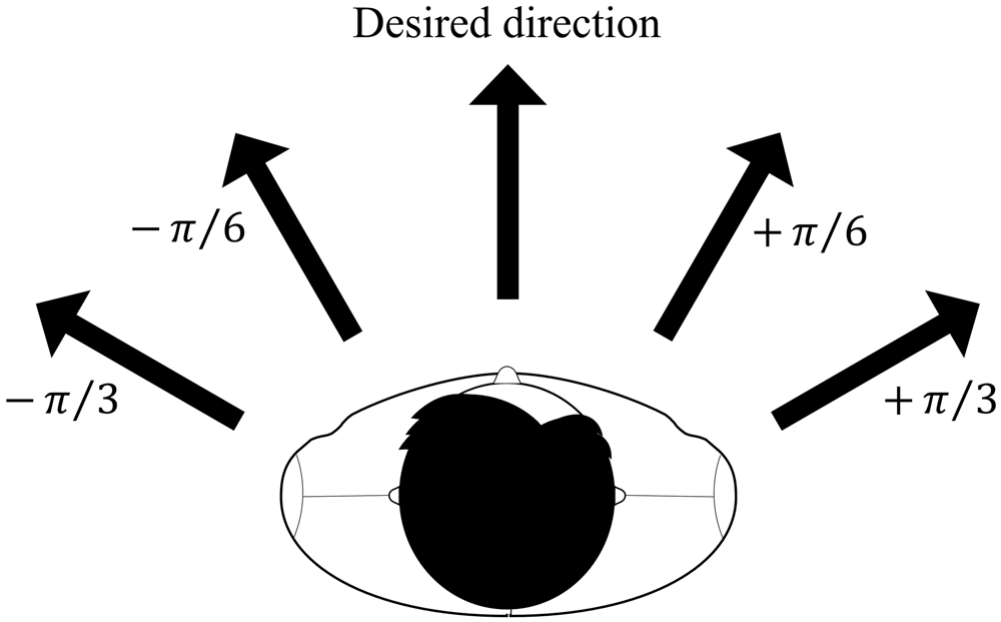
Area division for checking a critical density.

**Figure 10:**
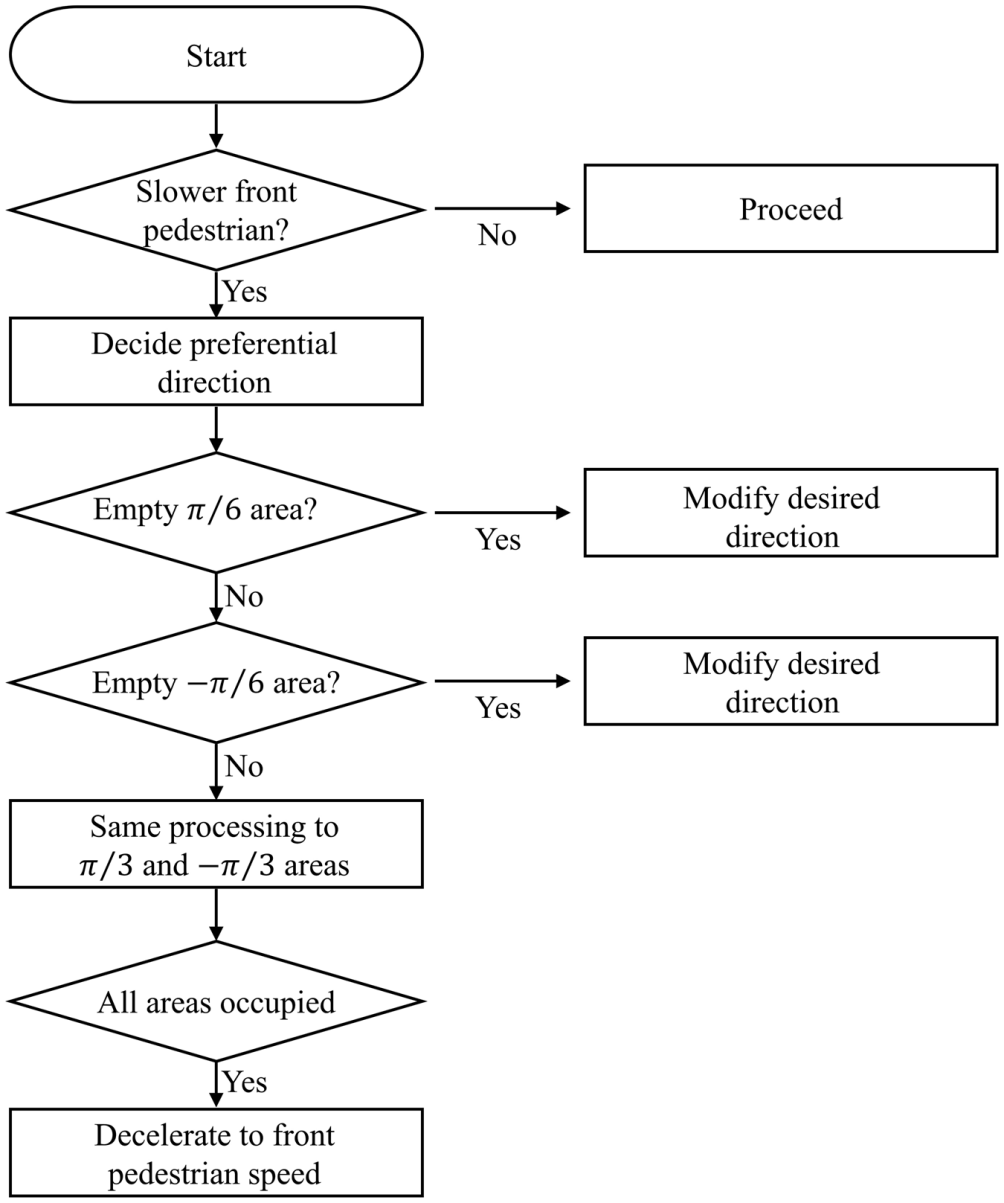
Decision making algorithm.

These simple rules are derived from the behaviour of a human crowd. However, a fairly similar movement of the mice can be observed in the experiment videos. An agent decides whether it overtakes the agent in front by comparing its current speed with the speed of the target. If the target is slower than the given agent, it tries to overtake the target by changing its desired direction. First, the given agent checks the area to the right $(+{\rm{\pi }}/6);$ if the density of the area is lower than a critical density (0.78 agent/${m}^{2}$ in the simulation), it can occupy that area. In a mouse model, the critical density is 0.77 mouse/$c{m}^{2}$. However, if there is someone in that area, the area to the left $(-{\rm{\pi }}/6)$ is considered. According to Scharine and McBeath^53^, the directional preference of people is related not only to their hand preference, but also cultural influences. Right-handers tend to consider the right side. When it comes to participants from different country, UK participants were less likely to choose the right side than US participants, who have right-dominant traffic rules. In addition, 85–90% of the human population are right-handed while the rest are left-handed^54^. Although we do not yet know if mice have hand preferences, we assume that they do, similar to humans. In this study, 90% of the agents in the simulation check the right side first and 10% of them check their left side first. The area division of pedestrians is shown in Fig. 9 and this direction changing procedure regarding the desired direction follows the flow chart given in Fig. 10. If all areas exceed the critical density, the given pedestrian slows, and subsequently follows the target.

#### Definition of a walking velocity

**Figure 11:**
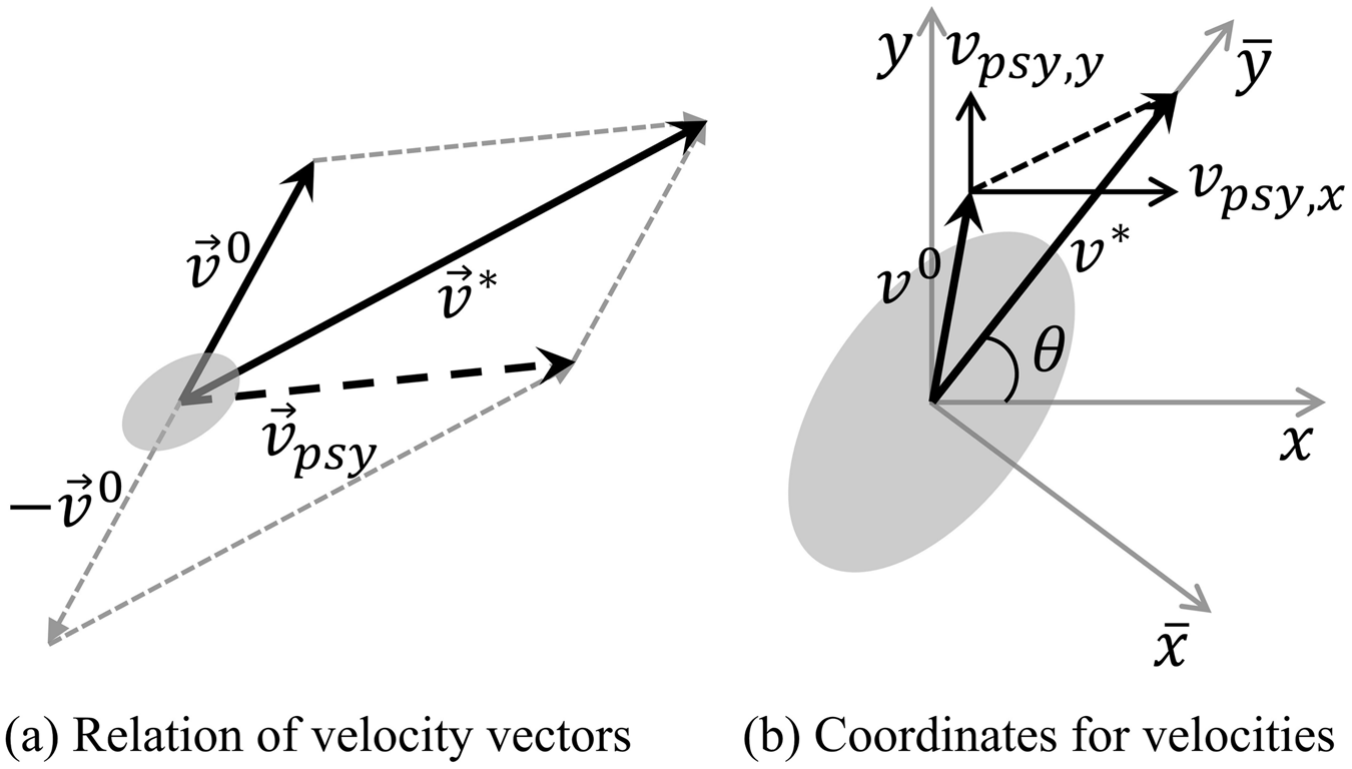
Current velocity and desired velocity.

The current velocity ${\vec{v}}^{0}$ and desired velocity ${\vec{v}}^{\ast }$ of each agent in the crowd cannot be the same. In such a case, agents try to keep up with the desired velocity by psychological velocity ${\vec{v}}_{psy}$, which also depends on the psychological situation mentioned above. Given that ${\vec{v}}^{\ast }={\vec{v}}^{0}+{\vec{v}}_{psy}$, as shown in Fig. 11, the psychological velocity can be represented as ${\vec{v}}_{psy}={\vec{v}}^{\ast }-{\vec{v}}^{0}$. Now that these values, consisting of vectors, are expressed by $\bar{x}-\bar{y}$ coordinates, the values should be transformed to *x* − *y* coordinate as follows,

4a $${v}_{psy,x}=\{({v}_{\bar{x}}^{\ast }-{v}_{\bar{x}}^{0})\sin \,\theta +({v}_{\bar{y}}^{\ast }-{v}_{\bar{y}}^{0})\cos \,\theta \}$$

4b $${v}_{psy,y}=\{-({v}_{\bar{x}}^{\ast }-{v}_{\bar{x}}^{0})\cos \theta +({v}_{\bar{y}}^{\ast }-{v}_{\bar{y}}^{0})\sin \,\theta \}$$

5 $${\vec{F}}_{psy}=(m/{\rm{\Delta }}{t}_{res}){\vec{v}}_{psy}={\rm{c}}{\vec{v}}_{psy}$$

Acceleration ${\vec{F}}_{psy}$ is defined in Eq. 5 and is velocity divided by time step ${\rm{\Delta }}t$. Here, agents should gradually change their walking speed and direction because of inertia effects in a given moment. This inertia effect should be represented by the response time needed to change the direction or velocity of an agent. This response time, ${\rm{\Delta }}{t}_{res}$, is approximately 0.1 s^55^. Therefore, constant *c*, which indicates how rapidly the current velocity reaches the desired velocity, is 500 kg/s in this work. Now, ${F}_{psy,x}$ and ${F}_{psy,y}$ can be represented as follows,

6 $${{\rm{F}}}_{psy,x}=c\{({v}_{\bar{x}}^{\ast }-{v}_{\bar{x}}^{0})\sin \,\theta +({v}_{\bar{y}}^{\ast }-{v}_{\bar{y}}^{0})\cos \,\theta \}$$

7 $${{\rm{F}}}_{psy,y}=c\{-({v}_{\bar{x}}^{\ast }-{v}_{\bar{x}}^{0})\cos \,\theta +({v}_{\bar{y}}^{\ast }-{v}_{\bar{y}}^{0})\sin \,\theta \}$$

where $\theta$ is the angle of the desired velocity in the $x\,$ direction, ${v}_{\bar{x}}^{0}$ and ${v}_{\bar{y}}^{0}$ are the components of the current velocity in $\bar{x}$ − $\bar{y}$ coordinates, ${v}_{\bar{x}}^{\ast }\,$is the component of desired velocity in the $\bar{x}$ direction, but is assumed to be zero to express the desired velocity briefly, and ${v}_{\bar{y}}^{\ast }$ is the component of the desired velocity in the $\bar{y}$ direction, where its magnitude is the desired speed.

The final equations of an agent’s motion can be expressed as follows.

8 $$\frac{{d}^{2}x}{d{t}^{2}}=\frac{c\{({v}_{\bar{x}}^{\ast }-{v}_{\bar{x}}^{0})\sin \,\theta +({v}_{\bar{y}}^{\ast }-{v}_{\bar{y}}^{0})\cos \,\theta \}}{m}+\frac{{f}_{x}}{m}$$

9 $$\frac{{d}^{2}x}{d{t}^{2}}=\frac{c\{({v}_{\bar{x}}^{\ast }-{v}_{\bar{x}}^{0})\sin \,\theta +({v}_{\bar{y}}^{\ast }-{v}_{\bar{y}}^{0})\cos \,\theta \}}{m}+\frac{{f}_{x}}{m}$$

All equations derived for agents are also applicable for mice without major changes. To apply these equations to mice, only the parameters are changed.

### Simulation Condition

**Figure 12:**
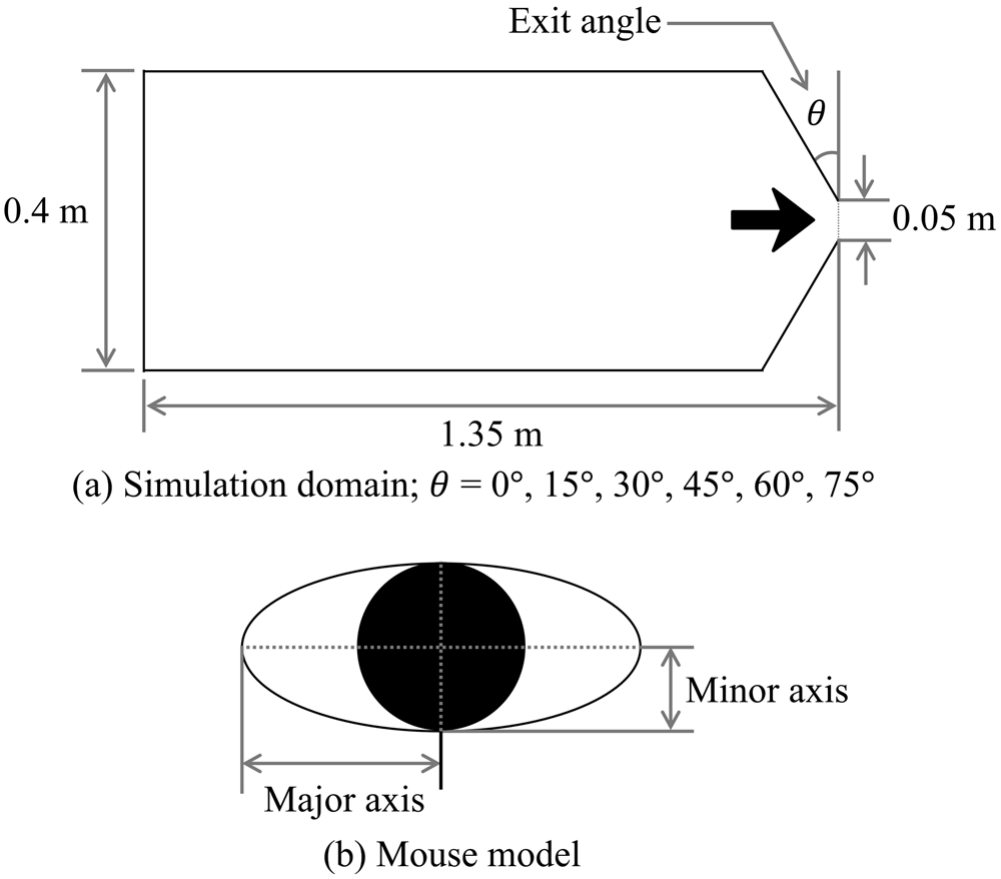
Simulation area and ellipse model for a mouse.

The experimental subjects in this research are mice; both computer simulation and experimental conditions should be the same to confirm the effectiveness of the custom code simulation. The parameter values are given in Table 1. Most values for the simulation, such as the desired velocity and standard deviation, are based on the experimental data. The remaining values, such as spring constant and friction coefficient come from other literature^23^. According to the configuration of the simulation, as depicted in Fig. 12, there is a single exit connected to guide walls at both sides, and the effect of these varied guide walls on the evacuation is observed.

**Table 1:** Simulation parameters.

Parameter	Value	Unit
normal spring constant	5.0 × 10^4^	N/m
tangential spring constant	5.0 × 10^4^	N/m
coefficient of restitution	0.1	
coefficient of friction	0.1	
calculation time step	1.0 × 10^−3^	s
mouse radius (in major axis)	3	cm
mouse radius (in minor axis)	1.5	cm
mouse weight	30	g

### Experimental Setup

#### Active avoidance test for rodents

There are several conventional mice behavioural tests: an active avoidance test, a passive avoidance test, the Morris water maze test, and a treadmill test^45^. In this research, the active avoidance test is adopted so that the mice will actively escape from a room where there is an obvious threat, just like in a real emergency. The threat-inducing panic situation can be modelled as fear conditioning with an aversive event: electric current delivered to a mouse through its feet. An Isolated Square Wave Stimulator (PHIPPS & BIRD, NO. 7092) applies the current; a shock intensity with an electric current ranging between 0 mA and 3.0 mA is given in the general behavioural test.

#### Mouse model

Eight-week-old mice, 25 male and 25 female C57BL/6N mice, were purchased from the Orientbio Inc. and bred at the *In Vivo* Research Center, Ulsan National Institute of Science and Technology (UNIST), South Korea. The C57BL/6N species is generally used in behavioural tests because of their genetic consistency. Their sexual maturity is achieved at an age of 6 weeks, their average weight ranges from 25 g to 30 g, and their width ranges from 2.5 cm to 3.0 cm.

#### Experimental procedure

**Figure 13:**
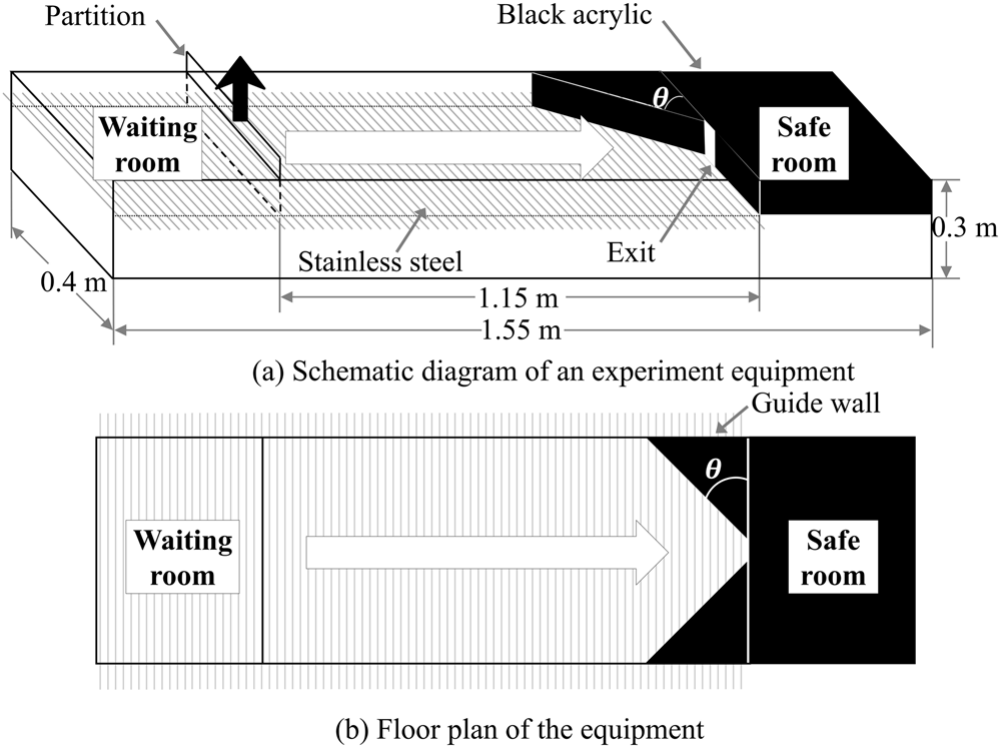
Equipment for the active avoidance test.

All experiments in this research were approved by the Institutional Animal Care and Use Committee (IACUC, authorized No.UNISTIACUC-16-21) and conducted at UNIST. In addition, authors of this article completed educational courses in research ethics and the experiments were performed in accordance with both the animal protection law and experiment protocol under the supervision of IACUC. The equipment for the active avoidance test is made of acrylic and stainless steel for electric stimuli. The equipment consists of three major parts: a waiting room, corridor, and safe room, as shown in Fig. 13. This equipment was used in a way similar to that in our previous research to verify the feasibility of rodent experiments^56^; the study has been enhanced and 50 mice are used, which is significantly more than in the previous research. First, the mice are confined in the waiting room separated from the corridor by a partition. After they receive 1.5 mA of electric current, the partition is removed, starting the evacuation. The panicked mice break for the exit through the corridor until they reach the safe room where the electric current is not present. Mice are known to feel comfortable in dark spaces to escape their predators; therefore, the safe room is dark. Various shapes of guide-walls are used throughout the experiment.

#### Extraction of data

After the entire process, the videos are analysed using ICY (ver. 1.9.1.0), an open source software for profiling mouse behaviour. Thus, information for the DEM simulation such as the average velocity of each subject, total average velocity of 50 subjects in all trials, total evacuation time, and standard deviation of the velocity are evaluated by software. Before we discuss the results, the definition of these terms should assist their understanding. The average velocity of each agent ${\bar{V}}_{t}$ can be written as follows:

10 $${\bar{V}}_{t}=\sum _{{\rm{\Delta }}t=0({t}_{i,start})}^{{\rm{\Delta }}t=n({t}_{i,exit})}{V}_{{\rm{\Delta }}t}/n$$

where ${V}_{{\rm{\Delta }}t}$ is the instantaneous velocity at ${\rm{\Delta }}t$, and *n* is the number of measurements. That is, the number of ${\bar{V}}_{t}$ should be 50 in a single case if there are 50 mice. If so, the average velocity of all agents ${\bar{V}}_{t,N}$ can be represented as

11 $${\bar{V}}_{t,N}=\sum _{i=1}^{i=N}{\bar{V}}_{t}/N$$

where *N* is the number of agents, which is 50 in this experiment. One of the major values we mainly discuss is the average ${\bar{V}}_{t,N}$ of all cases per each exit angle ${\bar{V}}_{t,N,C}$, given by

12 $${\bar{V}}_{t,N,C}={\bar{V}}_{total}=\frac{\sum _{i=1}^{i=C}{\bar{V}}_{t,N}}{C}$$

where *C* is the number of cases per each exit angle. In this research, three cases per each exit angle are recorded because attempting an excessive number oexperiments could lead to subject health problems, which is unethical. In addition, ${\bar{T}}_{evc}$, the total evacuation time of all cases per each exit angle is written as follows:

13 $${\bar{T}}_{evc}=\sum _{i=1}^{i=C}{T}_{evc}/C$$

where ${T}_{evc}={T}_{lastpedestrian,exit}-{T}_{firstpedestrian,start}$. There are similar terms regarding each agent’s evacuation time. The time to complete the evacuation oeach agent ${t}_{evc}$ is given by

14 $${t}_{evc}=\sum _{i=1}^{i=N}{t}_{{N}_{th}pedestrian,exit}-{t}_{{N}_{th}pedestrian,start}/N$$

and the average evacuation time ${\bar{t}}_{evc}$ is written as

15 $${\bar{t}}_{evc}=\sum _{i=1}^{i=C}{t}_{evc}/C$$

and can be determined from ${t}_{evc}$. Lastly, ${S}_{mice}$, the standard deviation of the velocity, is defined as follows,

16 $${S}_{mice}=\frac{\sum _{j=i}^{j=C}\frac{\sum _{i=1}^{i=N}\sqrt{{\int }_{0}^{t}{(\overline{{V}_{t}}-{\overline{V}}_{t,N})}^{2}}}{N}}{C}$$

This is is a double-averaged value with rpect to the number of agent and cases, thus it has different value according to the exit angle. Therefore, a uniform standard deviation of velocity ${S}_{uni}$ is given by

17 $${{\boldsymbol{S}}}_{{\boldsymbol{uni}}}=\sum _{{\boldsymbol{i}}=1}^{{\boldsymbol{i}}={\boldsymbol{E}}}{{\boldsymbol{S}}}_{{\boldsymbol{mice}}}/{\boldsymbol{E}}$$

where E is the number of exit angles, which is six in this work.

### Data Availability

The datasets generated during the current study are available from the corresponding author for reasonable requests.
